# Plant chemical defense allocation constrains evolution of tolerance to community change across a range boundary

**DOI:** 10.1002/ece3.657

**Published:** 2013-10-05

**Authors:** David H Siemens, Riston Haugen

**Affiliations:** Biology, Integrative Genomics Program, Black Hills State UniversitySpearfish, South Dakota, 57799

**Keywords:** Ecological defense costs, glucosinolates, plant community, range limits

## Abstract

Because transplant experiments show that performance usually decreases across species range boundaries, some range limits might develop from factors and processes that prevent adaptation to stressful environments. Here, we determined whether an ecological cost of plant defense involving stress associated with changes in the local plant community may contribute to range limit development in the upland mustard species *Boechera stricta*. In a common garden experiment of 499 *B. stricta* plants, performance decreased and a multivariate axis of community structure increased across the boundary, indicating increased stress associated with the community change. There was also significant genetic variation (evolutionary potential) among marker-inferred inbred lines of *B. stricta* for tolerance to the stress; however, lines with high basal levels of glucosinolate toxins had lower tolerance to the change in community structure. We suggest that defense allocation, which is also needed across the range, may impede adaptation to the stress associated with the community change and thus contribute to range limit development.

## Introduction

A central question in evolutionary ecology is what factors and processes contribute to the development of species range limits (Parmesan et al. 2005; Gaston 2009; Sexton et al. 2009; Wiens 2011). The importance of environmental factors to range limits comes partly from transplant studies, most of which show decreased performance across range boundaries (Sexton et al. 2009). Consequently, adaptation may be required to occupy stressful areas across range boundaries. Major factors that may prevent the process of adaptation across range boundaries include swamping gene flow from the center of the range, lack of genetic variation in range margin populations, lack of adequate time for adaptation, or dispersal barriers (Sexton et al. 2009). However, even when these factors are conducive to adaptation, another set of factors associated with genetic architecture, including multiple interacting loci and pathways, may result in genetic, physiological, or developmental constraints (trade-offs) that impede adaptation and the evolution of range expansion (Kawecki 2008). But what traits and underlying physiological and genetic mechanisms might be involved in such trade-offs and whether these constraints are important remain largely unknown (Etterson and Shaw 2001; Westoby and Wright 2006; Angert et al. 2008; Donovan et al. 2011).

Here, we tested for a trade-off that may contribute range limit development in plants at low elevation range boundaries where multiple biotic environmental factors may be important, such as herbivory and competition (e.g., Ettinger et al. 2011). We studied *Boechera stricta* (Brassicaceae), a close wild relative of *Arabidopsis thaliana*, in a low elevation and isolated region at the eastern edge of its geographic range. Specifically, we determined whether there was a negative genetic correlation between chemical defense allocation and tolerance to stress associated with the change in community structure across the range boundary. In general, this trade-off would be considered an indirect cost of defense allocation, also called an ecological defense cost. Many studies have documented direct “costs of production” for defensive compounds (Strauss et al. 2002), which is detected as decreased fitness in defended populations in the absence of consumers (Simms and Rausher 1987). The notable defensive compounds in mustard plants include glucosinolate (GS) toxins (Hopkins et al. 2009). Direct costs of GS production have been detected (e.g., Siemens et al. 2002), even in *B. stricta* (Siemens et al. 2010); however, ecological costs of defense are thought to be more important and frequent because decreases in plant fitness of defended populations are more readily detected under uncontrolled environmental conditions (Koricheva 2002 for review), suggesting that interactions of plants and their environment (competitors, pollinators, different types of herbivores, their natural enemies, and various types of abiotic stressors) are important for defense trade-offs.

We have previously documented a similar trade-off in *B. stricta* between GS allocation and general stress tolerance associated with a low elevation range boundary (Siemens et al. 2009), but the trade-off was only detected during a below average dry year, and drought stress in the laboratory was sufficient to induce the evolutionary trade-off (Siemens et al. 2012). Because drought stress may be an important ecological gradient for many low elevation range margin populations, we concluded that the trade-off may be a contributing factor to low elevation range limit development. However, our transplant experiments have also shown that the area outside the range can still be stressful even in years with above average precipitation (Siemens et al. 2012), suggesting that other stressors, such as competition, might also be present and important in a trade-off with defense allocation.

For a system in which one would expect adaption along important ecological gradients for range expansion, we show evidence that the process of adaptation to stress associated with the change in community structure across the range may be inhibited by an ecological cost of chemical defense. This result is in contrast to theory in evolutionary ecology, which predicts that (1) biotic factors are less important than abiotic factors to limit range, (2) any role of neighboring plants to limit range involves direct effects of resource limitation, and (3) plant defenses may be evolutionary responses to spatial distributions of plants and correlated life history traits (Siemens et al. 2009). Furthermore, we discuss an underlying mechanism for the ecological cost that involves selection acting on antagonistic defense and stress tolerance signaling pathways, and we provide supplementary evidence implicating abscisic acid signaling in the stress response.

## Methods

### Study organism and field site

*Boechera stricta* is a genetically diverse short-lived perennial that ranges at higher altitudes (e.g., 2500 m) across mountainous regions of western North America (Song et al. 2006, 2009). We studied *B. stricta* at its eastern geographic range boundary in a geographically isolated low elevation mountain range, the Black Hills of South Dakota. Black Hills populations of *B. stricta* may also be genetically isolated from populations in the Rocky Mountains located at similar latitudes (Fig. S1). Other genotype data suggest that *B. stricta* in the Black Hills originated from lower latitudes in the southern clade of *B. stricta* (Song et al. 2009; Lee and Mitchell-Olds 2011), which may help explain the occurrence of *B. stricta* in the Black Hills, where altitudes are much lower (e.g., 1700 m) than altitudes usually inhabited by *B. stricta* (T. Mitchell-Olds, pers. comm.). Probably as a consequence of marginal habitats in the Black Hills, plants of *B. stricta* in the region occur in isolated patches on more north-facing inclines and where adjacent trees provided additional shade. The experimental area just a few meters across the diffuse local range boundary studied was on a more south-facing incline and represented a more stressful environment for *B. stricta* (see Results), probably because it was less shaded from trees, lower in major soil nutrients (probably as a consequence of significantly less leaf litter), higher in attack rates by generalist insect herbivores (e.g., several species of grasshoppers that prefer more open and dry environments at lower elevations [Wachter et al. 1998]), and more dense with at least one interspecific neighboring plant species, as documented previously (Siemens et al. 2009).

### Marker-inferred lines

Using structure analysis (Pritchard et al. 2000) on data from seven polymorphic microsatellite markers, we previously identified six putative naturally occurring inbred lineages (hereafter lines) from a haphazard sample of 243 *B. stricta* plants from the study area (Siemens et al. 2009). When we established the common garden experiment in 2008, we planted several replicates of seven sib families from each of the lines to make sure that we had full representation of the genetic variation and thus evolutionary potential that occurred in the area. Sib-families within a line are more similar to one another than are sib-families from different lines because the breeding system of *B. stricta* is predominantly self-fertilizing (Roy 1995; Song et al. 2006, 2009). Full-sib families and lines were used because for self-fertilizing species, natural selection acts on total genetic variation (additive and nonadditive) and not exclusively on additive genetic variation (Conner and Hartl 2004, p. 108). In this study, we focused on variation among the lines because community structure of neighboring plants was so variable that even with about 500 *B. stricta* plants in the common garden experiment, there was insufficient replication within sib families for reliable estimates of the range of community structure encountered by each sib-family.

### Candidate traits

Stress tolerance was measured as the slope of a reaction norm of plant performance across a stress gradient (Simms 2000). The gradients in this study were the multivariate environmental gradient associated with the range boundary in the field, and the specific gradient represented by the change in neighboring plant community structure across the range boundary. Genetic variation in stress tolerance could therefore be detected by a significant line-by-gradient interaction in an analysis of covariance (ANCOVA) of plant performance. Plant performance was measured as basal rosette size (width), which is correlated with over-winter survivorship across the range boundary in the field (Siemens et al. 2009). Although survivorship across the range is too low for measures of reproduction or other life history analyses, shoot size is also correlated with reproduction across ecological gradients such as drought in the lab (regression: *F*_1,308_ = 37.073, *P* < 0.001, *r*^2^ = 22.7%). Thus shoot size, which is also correlated with root weight in the lab across ecological gradients such as drought (D. Siemens, unpubl. data, *r* = 0.381) is a reliable surrogate for plant size and correlated survivorship, which are appropriate measures of fitness in this case. The common garden experiment was planted in the fall of 2008 and the performance data for this study was collected in the spring of 2010, representing over one full season of growth in the field.

GS can have negative effects on a number of natural enemies, including pathogens, generalist insect herbivores, and interspecific neighboring plants (Siemens et al. 2002; Lankau 2008; Schlaeppi et al. 2008; Bednarek et al. 2009). Basal GS values were measured by high-performance liquid chromatography (HPLC) from plants in a previous generation grown in a growth chamber, as described elsewhere (Haugen et al. 2008; Siemens et al. 2009). Because of the self-fertilizing breeding system, sib-family or line mean GS values are similar across generations. Although environmental effects on GS can be expected, we have found that the basal levels can be useful to understand genetically correlated effects important for evolutionary inferences in the laboratory and field.

### Common garden experiment

The split-plot design of the common garden experiment is described elsewhere (Siemens et al. 2009). Briefly, three replicates of 42 full-sib families, representing all six marker-inferred inbred lineages, were planted in a randomized design in several blocks within and across the range boundary. We planted 20 blocks total, 10 located inside the area of natural occurrence and 10 just 30 m across the range boundary. Seedlings were started in the laboratory and then transplanted to the field in the fall after a brief acclimation period. We used seed from plants reared for one generation in a common growth chamber environment to minimize any differences in environmental maternal effects among lines and among families. Experimental plants in blocks were spaced at 10 cm centers within the diverse and dense meadow community. Thus, the nearest neighbors of the small *B. stricta* plants in the experiments were other species of plants rather than con-specifics, just as occurs naturally.

### Plant community structure

**Figure 1:**
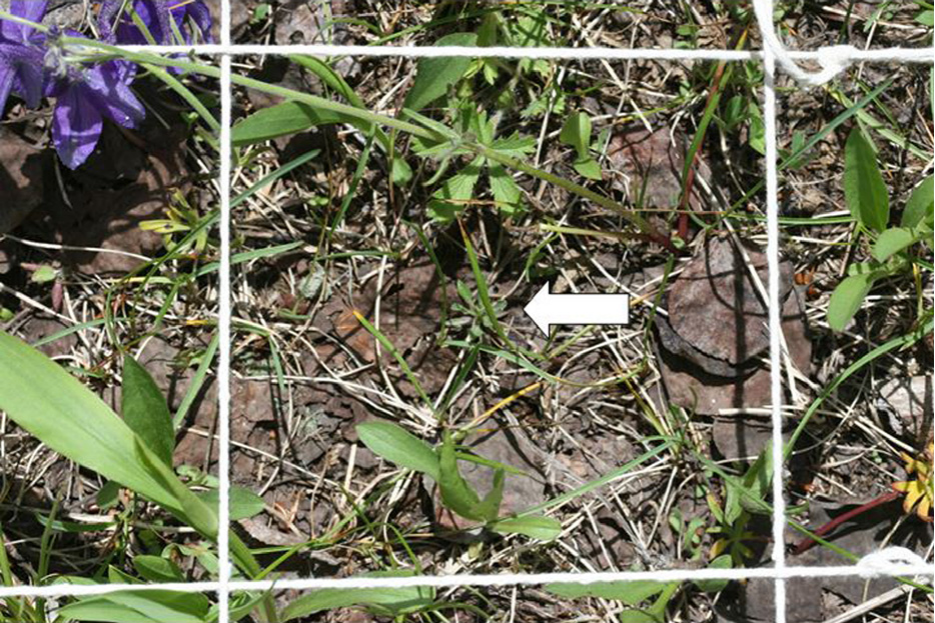
Basal rosette of a *Boechera stricta* plant surrounded by a relatively sparse plant community in the common garden experiment. The census grid square is 100 cm^2^.

Within 100 cm^2^ of each small experimental *B. stricta* plant in the common garden (Fig. 1), we recorded the number of individuals of each neighboring plant species, which were also relatively small, for a total sample size of 499 communities. We recorded over 37 neighboring plant species (Table S1); therefore, we used ordination techniques to reduce the dimensionality of the plant community data, as is often done in the analysis of plant community structure (McCune and Mefford 2011). That is, instead of analyzing the variance of each neighboring species separately, we analyzed a few orthogonal composite axes of variance. Specifically, we were interested in the ordination axis that changed significantly across the range boundary as a measure of the relevant change in community structure. We used nonmetric multidimensional scaling (NMS) in PC-ORD Version 4 (McCune and Mefford 1999) initially, but we switched to principal component analysis (PCA) in SYSTAT 13 after finding that either ordination technique yielded the same result, that the second ordination axis varied significantly across the range boundary (see Results). The ordination axes from NMS and PCA analyses were correlated (e.g., *r* = 0.419), and subsequent analyses were more readily conducted in SYSTAT 13. PCA is not usually suited for plant community data because of problems with skewed data distributions caused by uncommon species (McCune and Mefford 1999), which was avoided here with the large sample sizes (about 500 communities) in a relatively small region where the same species were repeatedly encountered and with the pooling of very small rare species.

### Detecting genetic variation in stress tolerance

To determine whether there was significant genetic variation in tolerance to the stressful area across the range boundary, and more specifically to the stress represented by the change in community structure across the range boundary, a full split-plot analysis was conducted using the following general linear model:


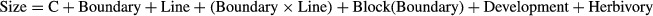
(1)

where Size is rosette size, C is a constant, Boundary is the boundary of natural occurrence (within the range and just across the boundary), Line is marker-inferred inbred lineage, “Block(Boundary)” is block nested within the boundary factor, Development is initial seedling size, and Herbivory is cumulative area (mm^2^) of leaf tissue consumed over the previous season. The whole-plot and within-plot factors that distinguish split-plot experiments were Boundary and Line, respectively. Genetic variation in stress tolerance was indicated by a significant Boundary-by-Line interaction in the analysis of plant performance (size). As also noted above, rosette size in this system is correlated with survivorship and reproduction and therefore represents a measure of fitness. The blocks controlled for any unmeasured random whole-plot factors not explained by the range boundary. Interaction between the within-block factor and block (Line-by-Block(Boundary)) was eliminated from the statistical model to simplify (Montgomery 1997), which did not affect our ability to detect effects of interest. Interaction terms involving the covariates, initial plant size, and herbivory were not significant (*P*'s > 0.1) and not of interest and therefore were also eliminated from the model. *F*-ratios for this split-plot model were correctly calculated for the random and fixed effects (Zar 1996; Montgomery 1997). All statistical analyses were conducted using SYSTAT 13.

A similar analysis was conducted to determine if there was significant genetic variation in tolerance to stress associated with the change in community structure, substituting community change for Boundary in equation (1), except the “Block(Boundary)” term remained the same. Stress associated with the change in community structure was measured along the plant community ordination axis that varied significantly across the range boundary.

### Detecting the trade-off

In the statistical analysis of genetic correlations to detect a trade-off, we used line means of rosette size after controlling for variation among blocks that was not explained by the boundary of natural occurrence (i.e., blocks nested within the boundary factor), and for development (initial seedling size). We did this by calculating means of residuals from an analysis that only included unmeasured random variation among blocks and initial size of seedlings.

## Results

### Effect of the range boundary on *B. stricta* performance

**Figure 2:**
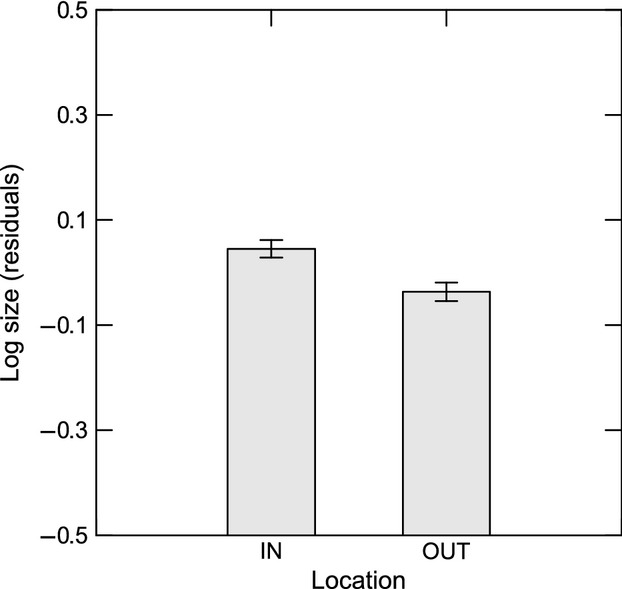
Size of *Boechera stricta* plants in the common garden experiment across the local range boundary from inside to outside the area of natural occurrence. Data are residuals of log-transformed size. The size data were log-transformed to satisfy assumptions of normality, and residuals control for random variation among flats and for variation in initial seedling size.

After more than a full year of growth in the common garden experiment, there was a significant difference in the size of the *B. stricta* plants across the natural boundary of occurrence (effect of boundary in the ANCOVA, *P* = 0.023, Table 1). Plants of *B. stricta* were in general about 21% smaller in the area just outside the normal range (Fig. 2); however, there was also significant genetic variation for tolerance to the stress (Line-by-Boundary interaction, *P* = 0.022, Table 1).

**Table 1:** The effect of marker-inferred inbred line and range boundary on *Boechera stricta* plant size achieved after 1.5 years of growth

Source	df	MSE	*F*-ratio	*P*-value
Line	5	0.048	0.480	0.792
Boundary	1,17	3.267	6.215	0.023
Line × Boundary	5	0.266	2.665	0.022
Block	17	0.526	5.263	<0.001
Development	1	1.646	16.480	<0.001
Herbivory	1	0.349	3.496	0.062
Error	423	0.100		
*r*^2^	26.3%			

### Patterns and apparent effects of plant community structure across the boundary

**Figure 3:**
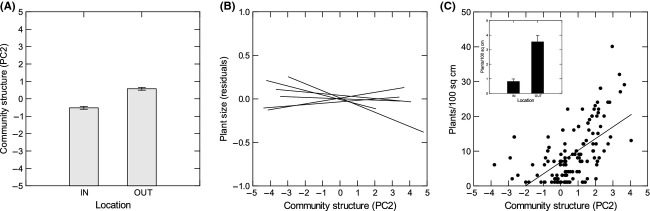
Community structure axis (second principal component – PC2) across the local range boundary from inside to outside the area of natural *Boechera stricta* occurrence (A). Variation among marker-inferred inbred lines of *B. stricta* for tolerance (slope) to the change in community structure (B). Relationship between the density of one of the potential competitors of *B stricta*, *Lithophragma parviflorum*, and the community structure axis – PC2 (C). Density of *L. parviflorum* across the local range boundary from inside to outside the area of natural *B. stricta* occurrence (inset). Note that the relationship between PC2 and glucosinolate allocation is in Figure 4.

Only the second principal component (PC2) varied significantly across the range boundary and with performance of *B. stricta* plants. The PCs were constructed from data on the abundance of neighboring plant species around each *B. stricta* plant in the common garden experiment. There were >37 species of neighboring plants recorded (Table S1). PCs that explained at least 1/37 or 2.7% of the total variance in the abundance of the neighboring species were considered for further analysis (Afifi and Clark 1984). The first six PCs each explained from 4.75% to 3.2% of the variance. Although an initial multivariate analysis of the effect of the range boundary on all six PCs was significant (*F*_6,492_ = 20.961, *P* < 0.001), in subsequent protected univariate tests (no Type I errors) only PC2 (*F*_1,497_ = 90.355, *P* < 0.001, Fig. 3A), PC5 (*F*_1,497_ = 15.825, *P* = 0.001), and PC6 (*F*_1,497_ = 16.754, *P* < 0.001) were significant. Of these three PCs that varied significantly across the boundary, only PC2 (Fig. 3A) had an apparent effect on performance of *B. stricta* plants, and this effect was dependent on marker-inferred line (Line-by-Community interaction in the ANCOVA, *P* = 0.019, Table 2, Fig. 3B).

**Table 2:** The effect of marker-inferred inbred line and neighboring plant community structure (second principal component – PC2) on plant size achieved after 1.5 years of growth

Source	df	MSE	*F*-ratio	*P*-value
Line	5	0.055	0.525	0.758
Community	1	0.477	4.523	0.034
Line × Community	5	0.290	2.747	0.019
Block	17	0.504	4.773	<0.001
Development	1	1.582	14.986	<0.001
Herbivory	1	0.182	1.724	0.190
Error	423	0.106		
*r*^2^	24.3%			

The change in community structure across the boundary measured as PC2 included the decrease in abundance of some species and increase in abundance of others. Neighboring plant species with relatively high influence on the axis of community change, PC2, included a species group that lumped grasses and sedges (component loading = −0.352; the negative sign indicating that species in this group decreased in abundance across the range boundary), prairie chickweed *Cerastium arvense* (Caryophyllaceae) (0.402), and prairie star *Lithophragma parviflorum* (Saxifragaceae) (0.446) (Fig. 3C and inset). The component loadings are correlation coefficients between the species abundance and the PC because the data was standardized before analysis (Afifi and Clark 1984).

In addition to the principal component analysis, we analyzed the plant community data using another ordination technique, NMS in PC-ORD, because plant community data are often highly skewed (McCune and Mefford 1999). But we found essentially the same results using both ordination methods; that performance across the range boundary was interdependent on inbred line and community structure. The first three NMS axes varied significantly across the range boundary (MANOVA: *F*_3,495_ = 17.287, *P* < 0.001). Again, as in the PCA analysis, the second ordination axis was the only axis that showed a significant effect on performance (Line-by-axis2 interaction: *F*_5,376_ = 2.609; *P* = 0.025).

### Effect of GS allocation on tolerance to the community change

**Figure 4:**
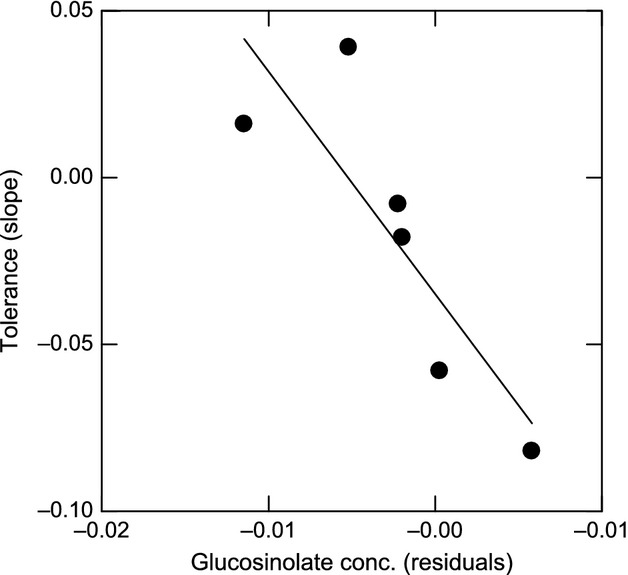
Relationship between tolerance to change in community structure (slopes of reaction norms in Fig. 3B) and the average concentration of the two common glucosinolates in *Boechera stricta*, 2-hydroxy-1-methylethyl and 1-methylethyl. Data are means of inbred lines, each determined by about 75 plants.

The relationship between tolerance to stress associated with the change in community structure across the range boundary and GS allocation was negative (Regression on line means: *F*_1,4_ = 10.170, *P* = 0.033, *r*^2^ = 71.8%, Fig. 4). Tolerance for each line was measured as the slope of the reaction norm of performance across the community structure gradient, PC2 (Fig. 3B). That is, tolerance measured how well a line performed against the change in plant community across the range. As the relationship was negative for each of the two major GS, 2-hydroxy-1-methylethyl and 1-methylethyl, we conducted the regression analysis on average GS concentrations. The average of the two GS for each line was calculated from residuals, which controlled for the potentially confounding factors of block and development. For one of the lines, instead of the average of the two GS, we just used the value for 2-hydroxy-1-methylethyl GS because the value for 1-methylethyl GS was identified as an outlier in the separate analysis. The outlier, however, is noteworthy because it was the line with the highest concentration of 1-methylethyl GS and also the highest tolerance value, which was just the opposite of the overall trend (Fig. 4). We used line means because the variance among the 499 plant communities was too large for analyses at the sib-family level. Furthermore, GS levels were only known for line or sib-family means because they were only measured in the laboratory.

## Discussion

Genetically diverse, abundant, range margin populations that are not inhibited by dispersal, or by swamping gene flow from the center the range or elsewhere, would be expected to adapt to stressful environments outside their range (Sexton et al. 2009); however, the existence of persistent range limits suggest that other constraints exist to prevent the process of adaptation from occurring in such cases (Kawecki 2008). The Black Hills represents a geographically and probably genetically isolated region at the eastern edge of the range of *B. stricta* where genetically diverse, low elevation populations face several abiotic and biotic challenges across local range boundaries, such as herbivory, drought, and competition (Siemens et al. 2009, 2012). Furthermore, the small winged seeds of *B. stricta* readily disperse across local range boundaries where soils do not inhibit germination (Siemens et al. 2012). Here, we investigated an ecological cost of plant defense, as defined above in the Introduction, which might be an important constraint on adaptation in range margin populations at low elevations where multiple biotic interactions are more common.

Because resource competition indirectly induces abiotic stress (e.g., from competitively low nutrient or water availability), and because we previously found that resource deficiencies are sufficient to induce the trade-off between defense allocation and stress tolerance (Siemens et al. 2012), one would predict that if the change in community structure across the range boundary represented resource competition, that the limiting resources would induce the trade-off. However, GS toxins may also confer a competitive advantage through allelopathic effects on competitors in some cases (Siemens et al. 2002; Lankau and Strauss 2007; Lankau 2008) thereby masking the predicted trade-off. Despite the potential for allelopathic effects, we found that allocation to GS was negatively genetically correlated with stress tolerance associated with the change in community structure across the range boundary (Fig. 4). We suggest that any allelopathic effects were minimal because we studied small, year-old plants that were transplanted as small seedlings into an established, mature natural plant community. That is, because of the competitive asymmetry in favor of many neighbors, any completive effects of *B. stricta* would be minimal. However, one outlier line with highest concentrations of 1-methylethyl GS also had high tolerance values, suggesting the potential for allelopathic benefits at high concentrations of some GS, but further tests are needed to verify any allelopathic effects in this system.

Alternatively, the increased density of some species of neighboring plants (e.g., *L. parviflorum*, Fig. 3C) may not indicate resource competition and may instead be correlated with other independent stressors. Several other potential stressors vary across the local range boundary studied (Siemens et al. 2009) including major soil nutrient availability, light, pH, disturbance, and limestone (CaCO_3_). But it should be noted that in the year of this study and the previous year, precipitation in the region was above average; therefore, drought and probably correlated temperature were not important stress gradients across the range boundary (Siemens et al. 2009). Needed are further experiments, such as removal of neighbors in the field, or laboratory experiments with competitors to test whether competition alone is sufficient to induce the trade-off.

We have suggested elsewhere (Siemens et al. 2009, 2012) that the evolutionary trade-off between chemical defense allocation and stress tolerance might be caused by selection at range boundaries acting on antagonistic signaling pathways. More stable expression of inducible stress tolerance and defensive traits is one way that plants may adapt to the stressful environments outside the range, but if there is negative crosstalk between the pathways, the simultaneous co-option of both pathways (tolerance and defense) for the evolution of range expansion may be constrained. We dubbed this the Defense Constraint (DC) hypothesis for range limit development. The idea comes from the well-substantiated work with Arabidopsis and other species showing cross-talk between defense and stress tolerance signaling pathways (Fujita et al. 2006; Asselbergh et al. 2008; Ton et al. 2009 for reviews) and from evolutionary genetic theory that predicts negative pleiotropy from such crosstalk in signaling networks (Des Marais and Juenger 2010). Specifically, abscisic acid (ABA)-mediated stress tolerance responses can take precedence over jasmonic acid (JA)- or salicylic acid (SA)-mediated defense responses. This cross-talk may be an adaptive switch if simultaneous responses are usually not needed. For example, in Arabidopsis, an ABA-mediated stress response under dry conditions takes 'precedence over JA- or SA-mediated defense responses that may function primarily against pathogens under moist conditions. Because range margin populations are thought to face challenges from both biotic and abiotic stressors, such as herbivory and drought, the cross-talk between defense and stress tolerance signaling pathways may prevent the simultaneous evolution of these traits that otherwise could occur via the co-option of the pathways for more stable expression outside the range.

We (Siemens et al. 2009) have also shown that attack by generalist herbivores is more frequent across the range boundary studied here and that 1-methylethyl GS reduced damage. However, for low elevation range margin populations, areas outside the range may also be drier and more diverse with potential competitors and therefore an increase in tolerance to these other stressors would also be needed for range expansion.

Circumstantial evidence implicating ABA signaling in the evolutionary trade-off (between drought stress tolerance and defense) has come from experimentation using exogenous ABA, which affected the evolutionary trade-off as one would predict if genetic variation in ABA and JA signaling mediated the stress response and the trade-off (Siemens et al. 2012). Soil injections of ABA changed drought tolerance responses, but the change was also dependent on genetic variation in basal GS levels. Might competitive interactions also elicit ABA signaling, as does drought, which would induce the trade-off? We have also grown *B. stricta* in the laboratory experimentally with two different putative competitors and then examined genome-wide gene expression of *B. stricta* plants using Arabidopsis microarrays (D. H. Siemens and R. Haugen, unpubl. data). One of the putative competitors, the goldenrod *Solidago missouriensis*, increased in density across the range boundary, while the other, dandelion *Traxacum officinale*, occurs with *B. stricta* in more disturbed microhabitats. When grown together with either putative competitor, *B. stricta* plants upregulated genes that had previously been implicated in response to dehydration, salt, or ABA treatments, thus implicating ABA signaling in the stress response to competition (Table S2).

Although species range limits are thought to be concordant with niche limits (Gaston 2009; Wiens 2011), biotic factors such as competition may be less important determinants of geographic range than abiotic factors (Wiens 2011). Instead, biotic factors may only be important determinants of local spatial distributions. However, empirical studies have found that abiotic factors and physiological limits are more important in extreme environments, such as at higher elevations, while biotic interactions, such as competition, may be more important within the range or at lower elevation range limits where there are more biotic interactions (e.g., Ettinger et al. 2011; but see Gifford and Kozak 2012). Theory also predicts that biotic interactions should be important when dispersal barriers and abiotic environmental gradients are weak (Case et al. 2005). In general, correlative studies examining the distribution patterns of potentially competing species more often (85%, *n* = 26 studies) supported a competition hypothesis for range limits (Sexton et al. 2009). Theoretical studies on competition and range development have mainly focused on effects of limiting resources (Price and Kirkpatrick 2009; Sexton et al. 2009). For example, theoretically, competition might limit range by: preventing colonization of occupied space beyond the range; causing the evolution of resource specialization; strengthening selection along a resource gradient; increasing gene flow asymmetry; or causing reproductive interference when hybrids are less fit. To the extent that the change in community structure that we measured represents competitive stress, to our knowledge, our study is the first to suggest that competition might limit range by inducing genetic constraints. To clarify, we found marker-inferred lines with inherently high basal GS levels were less able to tolerate the stress associated with the change in plant community structure across the range, and we assume that plant competition contributed to the stress.

## Supporting Information

**Figure S1.** Population genetic structure for individuals of *Boechera stricta* from the Black Hills (BH), the Big Horn Mountains (BHM) – another isolated mountain range at the eastern edge of the geographic range of *B. stricta* – and west in the Rocky Mountains (RM). We used 16 polymorphic microsatellite loci from Song et al. (2006) and STRUCTURE (Pritchard et al. 2000) as in Song et al. (2006), except that we used 1 million Markov chain Monte Carlo iterations and a burn-in period of 50,000. The RM samples were from representative sites in Idaho and Montana (Song et al. 2006) and were graciously provided by Drs. Bao-Hua Song and Thomas Mitchell-Olds. BH plants were located in the vicinity of 44° 24′50″ N, 103° 56′18″ W, elevation 1365 m, and BHM plants 44° 18′22″ N, 107° 18′33″ W, elevation 2780. Two of the samples (i.e., plants) assigned to the RM group by STRUCTURE were from BH (plants 3 and 9), but the lack of evidence of interbreeding between BH and RM individuals suggests that these individuals may be recent introductions.

**Table S1.** Species list of neighboring plants.

**Table S2.** In the laboratory, neighboring interspecific plants elicited ABA signaling in *Boechera stricta* plants as indicated by analysis of genome-wide gene expression using *Arabidopsis* microarrays.
