# Supplementary material for: Plant chemical defense allocation constrains evolution of tolerance to community change across a range boundary
Source: Ecol Evol. 2013 Oct 5;3(13):4339–47. doi: 10.1002/ece3.657 (PMC3856735; doi:10.1002/ece3.657)

Table A1. Species list of neighboring plants.

|  | Family | Genus | Species |
| --- | --- | --- | --- |
| 1 | Caprifoliaceae | Symphoricarpos | albus |
| 2 | Primulaceae | Androsace | occidentalis |
| 3 | Fabaceae | Trifolium | pratense |
| 4 | Caryophyllaceae | Cerastium | arvense |
| 5 | Scrophulariaceae | Collinsia | parviflora |
| 6 | Rosaceae | Potentilla | gracilis |
| 7 | Santalaceae | Comandra | Umbellate |
| 8 | Berberidaceae | Berberis | repens |
| 9 | Asteraceae | Antennaria | microphylla |
| 10 | Ranunculaceae | Delphinium | bicolor |
| 11 | Fabaceae | Vicia | americana |
| 12 | Liliaceae | Smilacina | stellata |
| 13 | Saxigrafaceae | Lithophragma | parviflorum |
| 14 | Ericaceae | Arctostaphylos | uva-ursi |
| 15 | Asteraceae | Solidago | missouriensis |
| 16 | Fabaceae | Trifolium | repens |
| 17 | Asteraceae | Taraxacum | officinale |
| 18 | Asteraceae | Antennaria | parviflora |
| 19 | Primulaceae | Dodecatheon | pulchellum |
| 20 | Asteraceae | Achillea | millefolium |
| 21 | Liliaceae | Zigadenus | venenosus |
| 22 | Liliaceae | Zigadenus | elegans |
| 23 | Scrophulariaceae | Verbascum | thapsus |
| 24 | Saxifragaceae | Heuchera | richardsonii |
| 25 | Oxalidaceae | Oxalis | dillenii |
| 26 | Fabaceae | Lupines | argenteus |
| 27 | Scrophulariaceae | Veronica |  |
| 28 | Violaceae | unknown |  |
| 29 | Lamiaceae | unknown |  |
| 30 | Rosaceae | unknown |  |
| 32, 33 | Bryophyta (unknown families) | grouped (two species) |  |
| 34-36 | small uncommon unknowns | grouped (three species) |  |
| 37- | Poaceae/Cyperaceae | grouped (several species) |  |

Table A2. In the ^‡^lab, neighboring interspecific plants elicited ABA signaling in B. stricta plants as indicated by analysis of genome-wide gene expression using Arabidopsis microarrays. For example, when grown next to Taraxacum, B. stricta up-regulated the genes listed below that were previously implicated in response to dehydration, salt or ABA treatments.

| Locus | Brief Description |
| --- | --- |
| AT1G73500 | Member of MAP Kinase Kinase family. |
| AT1G78080 | Encodes member of DREB subfamily A-6 of ERF/AP2 TF family |
| AT5G17310 | UTP--glucose-1-phosphate uridylyltransferase |
| AT5G47030 | Encodes the mitochondrial ATP synthase subunit delta. |
| AT5G49630 | Amino acid transporter |
| AT4G00430 | Member of the plasma membrane intrinsic protein subfamily PIP1. |
| AT4G17615 | Member of AtCBL (Calcineurin B-like Calcium Sensor Proteins) family. |
| AT2G45640 | Involved in the regulation of salt stress. |
| AT2G46830 | Encodes a transcriptional repressor |
| AT4G34000 | Encodes an ABA-responsive element-binding protein |
| AT5G15970 | Unknown protein; cold acclimation and salt tolerance. |

^‡^The competition treatments (next to Solidago, Taraxacum or alone) varied among 24 planting flats, each containing 15 pots (8 flats per competition treatment x 3 treatments x 15 B. stricta plants per treatment = 360 B. stricta plants total). Seed was from one inbred B. stricta plant to reduce genetic variation among individuals. Each sample consisted of entire shoots taken from 4-5 similar individual plants within a flat that were pooled for RNA extraction. Treatments were compared on *Arabidopsis* 70-mer oligonucleotide spotted whole-genome microarrays. Hybridization and target preparation was performed according to <http://www.ag.arizona.edu/microarray/> . The arrays were scanned using an Axon Instruments GenePix® 4200A scanner equipped with GenePix® v6.0 software. The resulting data files were imported into SAS, JMP® Genomics for statistical analyses. ANOVA was conducted on loess normalized log_2_ expression data. Genes with a log_2_ expression ratio of ≥ 1 or ≤ -1 that had an associated p-value of ≤ 0.1 were considered to be differentially expressed. A p-value threshold of ≤ 0.1 was chosen because this experiment involved cross-species hybridization and had a low sample size. Differentially expressed transcripts were categorized with respect to the biological process in which they are involved using the Gene Ontology (GO) annotation tool from TAIR .

Figure A1. Population genetic structure for individuals of B. stricta from the Black Hills (BH), the Big Horn Mountains (BHM) -- another isolated mountain range at the eastern edge of the geographic range of B. stricta -- and west in the Rocky Mountains (RM). We used 16 polymorphic microsatellite loci from Song et al. and STRUCTURE as in Song et al. , except that we used 1 million Markov chain Monte Carlo iterations and a burn-in period of 50,000. The RM samples were from representative sites in Idaho and Montana and were graciously provided by Drs. Bao-Hua Song and Thomas Mitchell-Olds. BH plants were located in the vicinity of 44^o^ 24’50” N, 103^o^ 56’18” W, elevation 1365 m, and BHM plants 44^o^ 18’22” N, 107^o^ 18’33” W, elevation 2780. Two of the samples (i.e., plants) assigned to the RM group by STRUCTURE were from BH (plants 3 and 9), but the lack of evidence of interbreeding between BH and RM individuals suggests that these individuals may be recent introductions.


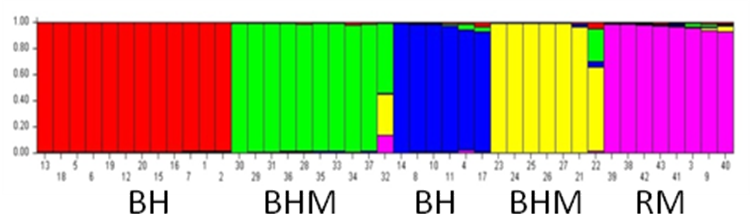

Supplement: Supplementary file 1 [file ece30003-4339-SD1.doc]
